# Do Children Copy an Expert or a Majority? Examining Selective Learning in Instrumental and Normative Contexts

**DOI:** 10.1371/journal.pone.0164698

**Published:** 2016-10-21

**Authors:** Emily R. R. Burdett, Amanda J. Lucas, Daphna Buchsbaum, Nicola McGuigan, Lara A. Wood, Andrew Whiten

**Affiliations:** 1 Centre for Social Learning and Cognitive Evolution, School of Psychology & Neuroscience, University of St Andrews, St Andrews, United Kingdom; 2 Centre for Ecology and Conservation, University of Exeter, Exeter, United Kingdom; 3 Department of Psychology, University of Toronto, Toronto, Canada; 4 School of Life Sciences, Heriot-Watt University, Edinburgh, United Kingdom; Centre for Coevolution of Biology & Culture, University of Durham, UNITED KINGDOM

## Abstract

This study examined whether instrumental and normative learning contexts differentially influence 4- to 7-year-old children’s social learning strategies; specifically, their dispositions to copy an expert versus a majority consensus. Experiment 1 (*N* = 44) established that children copied a relatively competent “expert” individual over an incompetent individual in both kinds of learning context. In experiment 2 (*N* = 80) we then tested whether children would copy a competent individual versus a majority, in each of the two different learning contexts. Results showed that individual children differed in strategy, preferring with significant consistency across two different test trials to copy *either* the competent individual *or* the majority. This study is the first to show that children prefer to copy more competent individuals when shown competing methods of achieving an instrumental goal (Experiment 1) and provides new evidence that children, at least in our “individualist” culture, may consistently express either a competency or majority bias in learning both instrumental and normative information (Experiment 2). This effect was similar in the instrumental and normative learning contexts we applied.

## Introduction

Children are committed social learners [1,2] disposed to encode and adopt the beliefs and skills displayed by others [3–13]. They may copy with great fidelity, but children can also be highly selective in whom they choose to copy [14]. This pervasive human capacity to learn socially and faithfully, yet selectively, is often cited as a reason for the magnitude and diversity of human culture compared to that of other species [2,15–17]. However, many studies that explore children’s copying fidelity and selectivity address unitary dimensions of choice, such as preferring to learn from a majority over a minority [4,7,10,18], or preferring to learn from relatively competent or knowledgeable others [19–22]. In real life, learning choices can be much more complex because there may be different reasons to favour alternative models. Other work has explored children’s selectivity across two dimensions. In particular, model competence seems to trump many dimensions, including age [23], native accent [24], and familiarity [25] but not others (e.g., group membership [26]). Perhaps the weighting that children place on model competence versus other dimensions depends on the learning goal. Accordingly in the current study we investigate children’s choices when two such influential dimensions are directly pitted against each other. Specifically, we aim to discover whether children choose among criteria concerning (i) majority preferences, versus (ii) model competence, by offering them a choice between learning the technique demonstrated by a competent individual versus the technique demonstrated by a majority of others, whose competence is unknown. We believe this is the first study to examine such a contrast in the acquisition of an action-related skill.

In addition, we investigate how such preferences may be influenced by a major contrast between what have been described as informational versus normative contexts, a classical distinction made some time ago in social psychology [27]. Informational (also described as instrumental) contexts are those in which the function of copying is to identify some correct information or useful adaptation to the real world, such as what things are best to eat or avoid or what is the best tool to use for a task. By contrast, normative contexts are those in which copying serves to align oneself with a behavior, belief, or value (or a cluster of behaviors, norms, or values) shared socially by a group, which in turn serves the function of facilitating social relationships. The latter may, for example, involve learning acceptable local customs or conventions, ‘taking a ritual stance’ [10], or simply strengthening affiliation through social similarity [28,29].

Researchers have theorized that children (and adults) may be expected to be biased by different social cues according to whether the learning context is either informational/instrumental or normative/conventional [3,10,12,30]. Hu and colleagues [12] conceptualised these as different “domain demands”. For example such contextual differences could be expected to matter when selecting whom to learn from. In an instrumental/informational context (e.g., solving a puzzle), choosing to learn from a highly competent individual could provide quality information to the person trying to learn a new skill, task, or behavior. By comparison, in a normative context (e.g., doing something how it “ought” to be done), the preferred testimony may be less clear. It is possible that copying a majority consensus may be the better way to learn a social convention. Normative behaviors are directly linked to knowledge that is transmitted through others, so conformity may be predicted to be an important factor for the spread and stability of such group-specific behavior, or cultural customs. However, it is also possible that children may prefer to copy from a more competent individual in any context.

We know from prior work that children will preferentially learn from the testimony of competent others (e.g., [22,31]), and also (separately) that children will prefer to copy a majority of a group (e.g., [4,7,10]). The latter studies have found that children will tend to copy a majority with higher fidelity when the context has a more normative or conventional frame [10,12,32]. However, other work has shown that there are limitations on children’s motivation to copy a majority. Children are less likely to copy a majority when the instrumental task is easier rather than more difficult [33], when the majority is unreliable or unsuccessful [6,34,35], and when the majority and the dissenter are both successful at achieving instrumental goals [12]. One question that these studies do not address is whether children will still copy a neutral majority (i.e., a majority where their prior success ─ or lack of success ─ is unknown) when they have the opportunity alternatively to learn from an expert, and whether instrumental versus normative contexts influence children’s selective trust in relation to such cues.

In this study, we bring together these topical questions concerning selective learning and trust with the phenomenon of cultural transmission through imitation. We contribute to a recently growing literature on selective learning, tool use and object manipulation [22,35–41]. We compared whether children are selective in whom they choose to copy (an expert—an individual who had demonstrated superior competence in similar tasks—versus a majority–with no previous history of competence) and whether this is influenced by normative versus instrumental contexts. We first tested experimentally whether children would choose to trust a more competent “expert” individual over a less competent individual in both instrumental and normative contexts. In a second, follow-up experiment, we employed a 2 x 2 design, exploring whether children prefer to copy an expert or a majority according to normative and instrumental contexts. In this way we explored the relative weight that children place on particular social cues (e.g., majority versus expertise) and whether children’s choices change according to normative versus instrumental contexts.

## Experiment 1: Discriminating between Competent versus Incompetent Models

In Experiment 1, we tested whether 4-to-7-year-olds selectively copy an instrumentally competent expert over an incompetent individual. We included 4-to-7-year-old children to examine whether there is a developmental progression in the degree of copying faithfully and whether there are differences in development according to context. We chose these ages because previous research has shown that older preschool and school-age children within this range show an increase in overall high fidelity imitation of social information compared to younger children [1,13,42,43] and children as young as four have shown that they are selective in choosing reliable individuals for information (e.g., [25,31]).

To test whether children selectively copy in different contexts, we used the same two, 2-action puzzle boxes in both instrumental and normative test conditions. We considered using a purer “normative” task, such as learning conventional table manners, and comparing this to an instrumental task, such as getting a prize out of a puzzle box. However, a design like this would not have allowed direct comparison across the two contexts. We used the puzzle boxes for retrieving rewards in the instrumental condition, and then attributed the same puzzle boxes with more normative dimensions in the normative condition. The instrumental condition was framed as affording the instrumental goal of getting prizes out of the puzzle boxes, whereas we added two normative cues in the normative condition. In this case we described the puzzle boxes as being for either “fepping” or “blicking”. Prior work has shown young children will interpret a task in a normative way when experimenters frame a task with such verbal cues (e.g. “this is how you dax” or “this is daxing”) [32,44–47]. Second, we showed children that there was a right or wrong way to operate the puzzle boxes, as demonstrated by social approval of one method and disapproval of another method. In addition to these cues we also removed the emphasis of an end goal by having no reward item in this normative condition [32] to avoid children seeing the task as instrumental. In addition, the experimenter did not gather the egg at the end of the demonstration, so that she would not appear to be doing the actions to gain the reward. The egg was left inside the opaque receptacle. Children did not receive a reward until the end of the experiment in the normative condition.

We chose to demonstrate the relative competence and incompetence of the two models, who would later act on the puzzle boxes, using instrumental tasks. Echoing the dilemma outlined above concerning the test contexts, we considered additionally contrasting a model competent in applying cultural norms with one incompetent in this respect, but feared such incompetence could likely be perceived as merely culturally strange or”weird” to the child. We think that this is a fundamental conundrum in this domain of research and decided to reject this approach, at least for the present study.

We additionally examined whether children who prefer to copy the expert, copy the actions they see more faithfully in the normative condition. Based on prior work [32], we predicted that normative actions will be more faithfully produced than instrumental ones, since they are performed as an end in themselves, rather than to achieve a goal.

### Methods

#### Ethics statement

Ethical approval for both studies was obtained through the University Teaching and Research Ethics Committee (UTREC) at the University of St Andrews (protocol PS10659). Parents of children provided written informed consent and all children provided verbal consent.

#### Participants

Forty-four children (*M* = 72 months, range = 49–93, 27 girls and 17 boys) participated. Twenty-two children were assigned to an instrumental condition (*M* = 74 months, range = 50–93, 13 girls and 9 boys) and 22 to a normative condition (*M* = 71 months, range = 49–93, 14 girls and 8 boys). An additional three children were tested but not included due to technical/experimenter error (n = 2) or shyness (n = 1). All children were recruited while they were visiting RZSS Edinburgh Zoo, and were tested in a secluded and quiet area covered by a large gazebo.

#### Design and procedure

In recruitment, children visiting the zoo with their parents were asked if they would like to have a look at puzzles we had brought to the zoo. Following parental consent, participating children were then allocated to either the normative or instrumental condition. The study then proceeded in three phases (for the script, see S1 File). In the first, History Phase, children in both conditions watched as two young women who acted as models were introduced via video presentation on a laptop screen. We used videos so that all demonstrations were consistent, including counterbalancing, and also for practicality of minimizing the number of persons involved, given that each child participant was to witness multiple models in the follow-on Experiment 2. One model demonstrated competence in solving three wooden puzzles (i.e., retrieving small prizes), and the other demonstrated relative incompetence (i.e., not being able to retrieve the prizes). There followed a second, Familiarisation Phase, in which children in both conditions were familiarised with the kind of test box they would later experience in the third, Test Phase. In the Familiarisation Phase they learned that one action was the “right” or successful action on a training puzzle box and another was the “wrong” or unsuccessful action. In the instrumental condition, a plastic egg containing a sticker was released into a side-compartment that was opaque. In the subsequent Test Phase, this opaque compartment ensured there was no visible evidence of which of the two models’ actions had been successful. This meant that children could be selective only in relation to the model’s History Phase. In the Test Phase children were reintroduced to the models. Each model applied a different action to each of two novel puzzle boxes in turn. After witnessing each model attempting to operate the first puzzle box on video, the puzzle box was placed in front of the child who was then allowed a turn. This sequence was repeated with a second puzzle box.

After the test trials, children were asked follow-up questions, inviting them to explain why they had chosen the options they performed, and checking they could remember how each model had performed in the history phase.

**Fig 1:**
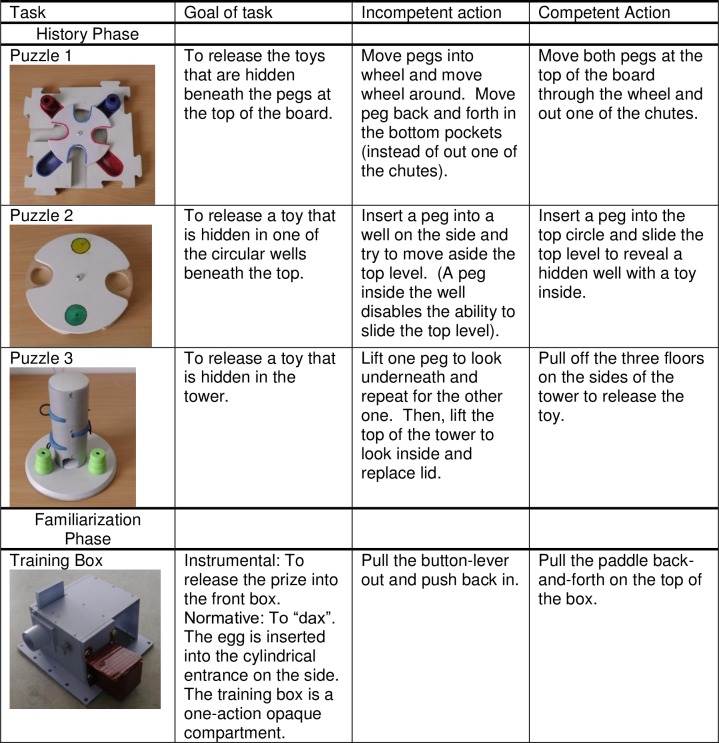
Tasks used in the history and familiarization phase of experiment including the goal and actions for each.

**History Phase:** Children sat in front of an 18” computer monitor. Via a presentation in Microsoft Power Point, children were introduced to two unfamiliar female models, clearly distinguishable by coloured shirts, and were told by an experimenter that they were going to watch both models take turns with three different puzzles. In total, children watched six videos. For each puzzle, children watched one video of a model with one shirt colour act relatively incompetently and try unsuccessfully to retrieve a prize from a wooden puzzle, and another video of the other model act competently and extract the prize successfully. This was repeated for three different wooden puzzles (see Fig 1). The order and the identity of the two models were counterbalanced.

After children watched both models work on the first puzzle, they were asked “which model got the prize out?” This question served to check that the children could correctly identify the competent model. If the child responded that the incompetent model retrieved the prize, the videos were replayed and the child was again asked which model got the prize out. The same process was repeated for the subsequent two puzzles. All but one child correctly identified the expert in all three history phase tasks. This child misidentified the expert in only one of the three trials. After re-watching both videos for the one test trial, this child correctly identified the more competent model and in the subsequent two history phase trials, correctly identified the expert.

**Familiarisation Phase:** Children in both instrumental and normative conditions were shown the same training box. This training box was of a similar shape to the test trial boxes so that children would become accustomed to these novel puzzle boxes. Unlike the test trial puzzle boxes (which had two actions that were both functional), the training box had two actions, but only one functional solution. One method did not work (pulling a lever out and back in). The other method, wiggling a paddle on top of the training box, released the egg into a smaller, opaque, lidded compartment (the shaded box shown in Fig 1). This opaque compartment was included so that children (in the instrumental condition) would become accustomed to the idea that acting on a box would not immediately reveal the egg if it had been correctly released. The purpose of this phase was to demonstrate that there were successful and unsuccessful (instrumental condition) or “appropriate” and “inappropriate” (normative condition) ways to operate a puzzle box. In both instrumental and normative conditions children learned of an unsuccessful or “wrong” way to operate the box (pulling a lever out) and a successful or “right” way (wiggling a paddle on the top). We expected that this experience would lead children to be more selective in their choice of models in the test trials, since they would not assume that both approaches would be favourable.

**(i) Instrumental condition:** The experimenter told the child that there was only one way to get a prize out. The experimenter dropped a prize (an egg with a sticker inside) through a chute in the top of the training box and first demonstrated one method and then asked the child to look inside the opaque compartment attached to the puzzle box to see if the egg and been released. The incorrect method was always shown first. Then the experimenter showed the correct method to the child and had him or her check the opaque compartment to find that the prize was inside.

**(ii) Normative condition**: Children were told that the training box was for “daxing” and emphasized that there was only one way to “dax.” The experimenter showed the child one possible way to “dax” (i.e. pulled out the lever) and then asked, “Was this the right way to dax? Let’s ask.” A video on the computer screen displayed frowning faces with thumbs down and auditory ‘boos’, communicating to the child through social disapproval that this was the wrong way to dax. The experimenter then demonstrated the alternative method (wiggling the paddle) and asked if this was the correct way to dax. The screen then displayed smiling faces with thumbs up and auditory applause, signalling to the child that this was the correct way to dax. To avoid having an instrumental end goal in this condition, we used an empty egg (i.e. with no sticker inside) and did not draw attention to where it had gone. The experimenter did not retrieve the egg at the end of the demonstration nor did children receive a reward until the end of the experiment. However we included an egg for methodological equivalency across normative and instrumental conditions.

**Fig 2:**
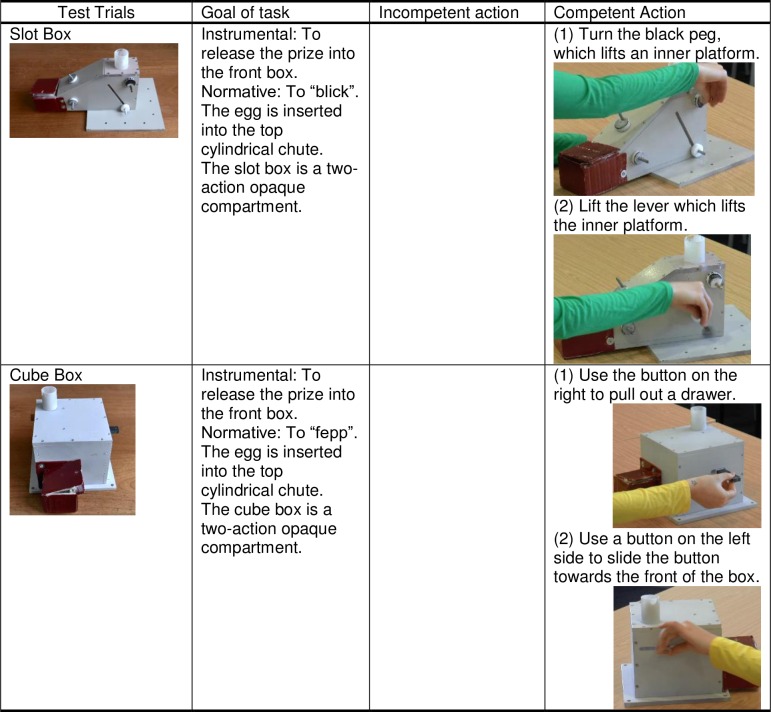
Tasks used in the test trials of experiment including the goal and actions for each.

**Test Trials:** In each of two test trials, children watched the two models from the History Phase in turn demonstrate a different method to operate two two-action puzzle boxes: the Slot box and the Cube box, the latter an adapted version of the “Sweep-Drawer” box used in an earlier study by Wood and colleagues [48]. Both boxes had a cylindrical chute through which a plastic egg could be dropped into the box. In each box two alternative manipulation sequences were effective in releasing the prize into an opaque compartment on the front, like that attached to the training box (Fig 2). Opaque compartments were padded with material so that children could not hear whether the plastic egg had been released into the box. Both the order of the test trials and of the models were counterbalanced.

**(i) Instrumental condition:** Children were invited to watch each model try to get the prize out. The model performed each action twice in succession to provide opportunity for variance in copying fidelity (e.g., pulling out and pushing back in the drawer twice). The video stopped at the point where the model was about to open the red prize receptacle, so that children did not see the outcome of whether the models in the test trials were successful (i.e., that their method released an egg). This is a novel and important part of the design, as the children knew the models’ history of success from the puzzles in the history phase, but did not know whether either model was successful on the novel test boxes. They were thereby encouraged to base their decision on prior history, avoiding revealing that the two methods were in reality equally successful. Following both videos of the two models, children were presented with the same puzzle box they had witnessed in the video and told it was their turn to get the prize out. This sequence was repeated with the second test puzzle box before children were allowed to open the opaque prize compartments and retrieve any eggs inside.

**(ii) Normative condition:** Children were presented with the first of the two novel puzzle boxes and told that it was for “fepping” or “blicking.” They then watched as each model on video performed one of the two alternative methods. As in the instrumental condition, each action was performed twice by the model. Then, children were told it was their turn to “fepp”/”blick”. This sequence was repeated with the second box. In the normative condition children had no need to open the opaque compartment following the end of the experiment since there were no prizes.

In both conditions the experimenter noted which action children chose, the number of times they repeated that action (that they had seen repeated twice by models), and whether the expert or incompetent model had demonstrated that action.

#### Follow-up questions

Following the test trials, the experimenter asked children two additional questions. First, children were invited to explain why they chose their method for each puzzle box. Second, they were asked to recall which model was “the best” at getting prizes out of puzzles. Children’s responses were later coded into a number of classes based on their content. Following coding, children’s responses were uninformative for understanding their choices, so we do not report them below. However, these responses are included in the supplementary materials (see S2 File and S3 File and S1 Table).

### Results

For each trial, children were given a score of 1 if they used the method that corresponded with the expert or a score of 0 if children used the method of the incompetent model. Scores were summed so they thus ranged from 0 (did not use the method of the expert in both trials) to 2 (used the method of the expert in both test trials) (S1 Dataset. Preliminary analyses showed that there were no order effects, no differences in performance between sexes and no difference in responses for the two test boxes (S4 File).

#### Whom did children copy?

**Fig 3:**
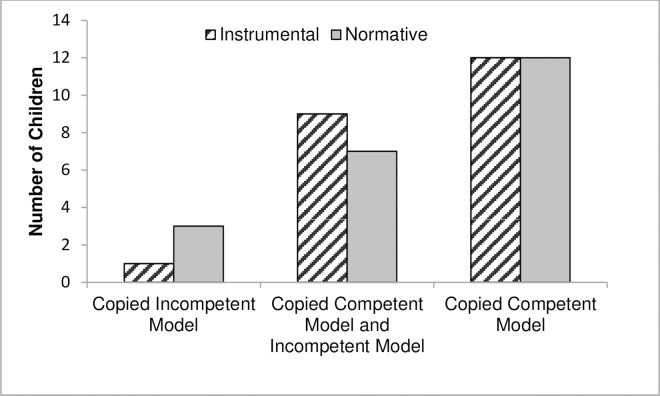
Number of children who copied the technique demonstrated by the competent or incompetent model in Experiment 1.

Overall, across the normative and instrumental conditions 24 children matched the method of the expert model on both test trials. An additional 16 children matched the method of the expert model in at least one test trial and just 4 children matched the method of the incompetent model in both test trials. Each child received a single classification of matching either (i) the competent model both times, (ii) the incompetent model both times, or (iii) both models once each. Chi-square goodness-of-fit tests against the expected chance distribution across these possibilities of 1:1:2 showed that children were more likely to match the method of the competent model than the incompetent model in both instrumental, *χ*^2^ (2, N = 22) = 11.73, *p* = .003, *r* = .73, and normative conditions, *χ*^2^ (2, N = 22) = 10.27, *p* = .006, *r* = .51 (see Fig 3). Binomial tests confirmed that the proportion of children’s competence responses (12 children in the instrumental and normative conditions) compared to children’s incompetence responses (one child in the instrumental and three children in the normative condition) were significantly different from chance in both the instrumental, 12 out of 13, *p* = .002, and normative conditions, 12 out of 15, *p* = .014. There were no differences in choosing the competent model between the instrumental and normative conditions, *χ*^2^ = 1.25, *p* = .62. We also explored whether age correlated with how likely children were to follow the method used by the competent model. There was no significant relationship for the instrumental condition, Pearson’s *r* = .07, *p* = .74, but children were more likely to match the method of the competent model in the normative condition with increasing age, Pearson’s *r* = .49, *p* = .022, *n* = 22.

#### Copying fidelity

**Fig 4:**
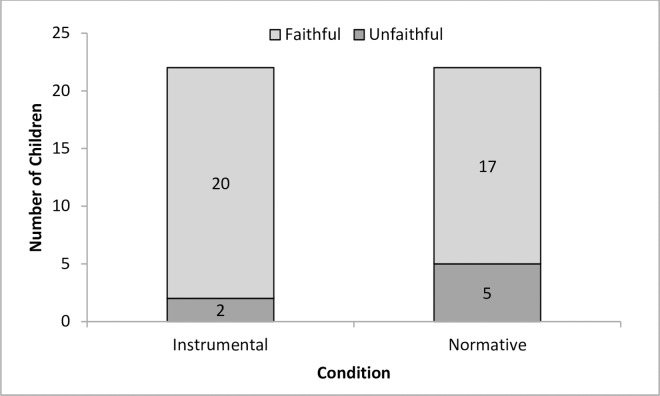
Number of children who copied the action faithfully and unfaithfully in both conditions in Experiment 1.

To examine how closely children reproduced what they saw, we coded children’s repetition of the actions (which in each case they saw repeated twice) in two categories: those who copied ‘unfaithfully insofar as they (copied the action only once, and those who copied the model ‘faithfully’ in that they repeated the action more than once. Our rationale was that in instrumental contexts a less faithful approach would suffice, whereas in normative contexts it should be more appropriate to achieve fidelity to the method witnessed (copying the action twice, like the model, or more). As in prior research we thus evaluated in this way how faithfully children copied, and if they did so more in a normative context. Using binomial tests, we found that across both test trials children were more likely to copy faithfully than copy unfaithfully in both the instrumental condition (20 children out of 22, *p <* .001) and the normative condition (17 children out of 22, *p =* .008, see Fig 4). There were no significant differences between conditions or test trials.

### Discussion

As predicted, results from Experiment 1 showed that children of all ages predominantly copy the technique demonstrated by an expert rather than a model earlier seen to be relatively incompetent. This result is consistent with previous research which has shown that children consistently trust more competent individuals [20,22,49]. On the basis of previous research we had predicted that children would copy the actions most faithfully in the normative condition, but we found that children tended to perform copies of the action significantly more than once, as the model had done, in both conditions. Because the actions were causally opaque, children were forced to remain unaware of how each method worked and we suspect that accordingly they may have tried to ensure they were acting effectively (with causal effectiveness more crucial in the instrumental condition) by repeating it, either exactly or even more than demonstrated.

This first experiment was conducted to establish whether children copy more competent models in both learning situations, which was confirmed. This was in preparation for a second experiment in which we tested the relative weighting that children place on competence versus other dimensions, dependent on the learning goal. We might expect children to prefer particular models based on social cues and the learning context. Specifically, in Experiment 2, we investigated whether children selectively trust a majority versus an expert model in normative versus instrumental contexts. We judged it unnecessary to run an experiment to test for copying of a majority per se, because such effects have been amply confirmed in similar, action-based research [7,10,18]. Because we found that older children were more likely to copy an expert in the normative condition and because previous studies have shown that older preschool and school-age children tend to imitate more faithfully [1,13,42,43], we collected a larger sample to include both a younger age group (4- to 5-year-olds) and an older age group (6- to 7-year-olds) to examine any developmental differences for how younger or older children select whom they choose to copy and whether imitative fidelity changes with age.

## Experiment 2: Discriminating between a Competent Model and Consensus

### Methods

#### Participants

In Experiment 2, we tested 40 4-to-5-year-olds, (*M* = 59 months, range = 48–71, 19 girls) and 40 6-to-7-year-olds, *(M* = 82 months, range = 72–94, 30 girls). Forty children (20 from each age group) were assigned to an instrumental condition and 40 children were assigned to a normative condition. As in Experiment 1, all children were recruited from a quiet location in the RZSS Edinburgh Zoo. An additional five children were tested but excluded due to technical/experimenter error (n = 1), shyness (n = 2), and because a caregiver prompted the child what to do in the test trials (n = 2).

#### Materials

We used the same history phase puzzles, training box and test boxes as in Experiment 1.

#### Design and procedure

**Fig 5:**
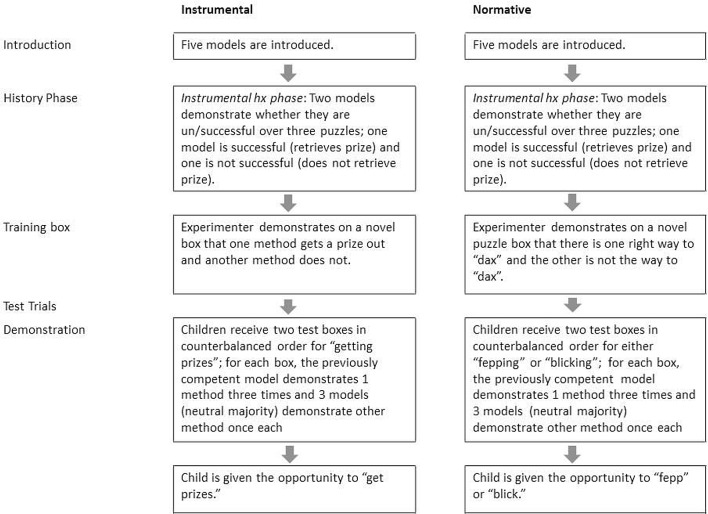
Design of Experiment 2.

Participants within each of the two age groups were randomly assigned to either an instrumental or normative condition. The experiment consisted of an introduction to the models, a history phase, and test trials (see Fig 5 for a schematic representation of the study and S5 File). The experimenter first introduced each child to five unfamiliar adult female models via a screen: three of these later appeared as the majority and two became the competent and incompetent models. The model’s identities were counterbalanced so that each had a turn at being an expert or part of the majority. All the models wore different, primary colored t-shirts. The experimenter introduced each model one-by-one while clicking on-screen which resulted in that model waving to the child.

Children were then presented with the same history phase as in Experiment 1, demonstrating which of two models was the more competent. At the end of the history phase children were asked who was best at getting prizes out of the puzzles. All but five children correctly identified the competent model in all three history phase trials. Each of the five children misidentified the expert model in only one of the three history phase trials. After re-watching both videos for that trial, all five children correctly identified the more competent model and in the subsequent two history phase trials, correctly identified the expert.

The incompetent model was not seen after these videos, as her role was only to help establish the competence of the expert model, while leaving the majority with unknown competence.

As in Experiment 1, children then experienced the familiarisation phase with the training box. In test trials, children then saw a total of 6 videos: the competent model demonstrating the same technique three times (3 videos) and three models (the majority) each demonstrating the alternative once (3 videos). As noted earlier, the structure required in such experiments was a significant factor in our decision to use video presentations rather than live models. Presentations of the actions of the majority and the competent model, as well as the order of the test trials, were counterbalanced (MMMCCC vs CCCMMM). After children watched the six videos, the experimenter reminded them what methods the majority and the expert used by pointing to six photographic stills presented on a laptop screen and saying which method the competent individual used and which method each of the three models of the majority used. To encourage equal reinforcement of both methods, three video stills showed the hands and method of the competent model and another three stills each showed the hands and method of each member of the majority.

### Results

Children were given a score of 1 if they used the method that corresponded with the expert or a score of 0 if they used the method of the majority. Scores were summed as in Experiment 1, so that 0 meant the child used the method of the majority in both trials and 2 meant they matched the expert in both test trials (S2 Dataset). If children chose the method of the majority in one test trial and the expert in the other, they scored 1.

#### Whom did children copy?

**Fig 6:**
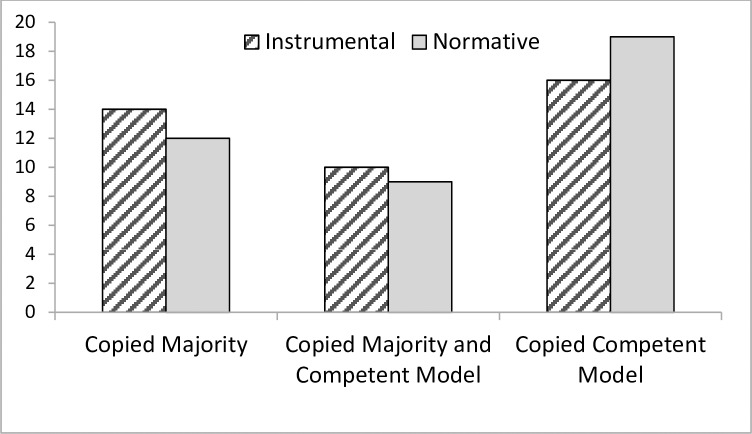
Number of children who copied the technique demonstrated by the competent model or the majority in Experiment 2.

We first examined whether children chose to follow the method performed by the majority or by the expert. Sixteen children in the instrumental condition and 19 children in the normative condition matched the method of the expert in both test trials. However an additional 14 children (instrumental condition) and 12 children (normative condition) matched the method of the majority in both test trials. Smaller numbers (10-instrumental, 9-normative) were ambivalent in matching the majority in one trial and the expert in the other. These distributions were significantly different from chance in both the instrumental condition, *χ*^2^ (2) = 10.20, *p* = .006, r = .51, and the normative condition, *χ*^2^ (2) = 14.55, *p* = .0007, r = .61, see Fig 6, indicating that children were significantly and consistently more likely to opt for one or the other strategy (a chance distribution would have the greatest number of matches in the central ‘1’ score). However Pearson chi-square tests revealed that there were no differences in the proportion of children who chose the majority or the competent model between conditions, *χ*^2^ (1) = .46, *p* = .71.

We also examined developmental differences and similarities among 4- and 5-year-olds and 6- and 7-year-olds. Across the two test trials, 6-to-7-year-olds were more likely to consistently copy either the majority *or* the expert than to be ambivalent in the instrumental, *χ*^2^ (2, *N* = 20) > 8.8, *p* = .02, and normative condition, *χ*^2^ (2, *N* = 20) > 7.5, *p* = .03. By contrast, 4-to-5-year-olds (*N* = 20) responded randomly in the instrumental condition, *χ*^2^(2) = 3.2, *p* = .21, but consistently chose one or other strategy in the normative condition, *χ*^2^ (2, N = 20) = 7.6, *p* = .023. However, chi-square tests of independence revealed no significant differences in children’s copying behaviors between age groups in either the instrumental, *χ*^2^ (2) = 1.01, *p* = .61, or the normative condition, *χ*^2^ (2) = .49, *p* = .78.

#### Copying fidelity

**Fig 7:**
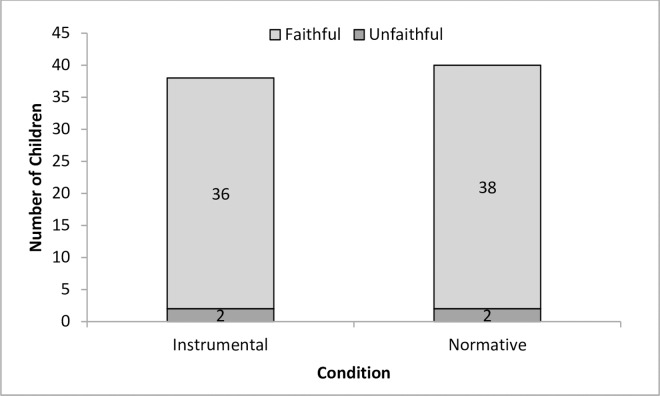
Number of children who over, under, or exactly copied the action in both conditions and test trials in Experiment 2.

We used binomial tests to examine relationships between instrumental and normative contexts and copying fidelity. As for Experiment 1, we examined whether children were more likely to copy the model unfaithfully (copy the action once) or faithfully (copy the action more than once) over both test trials and by instrumental or normative condition. Similar to Experiment 1, we found that children were more likely to copy faithfully rather than unfaithfully in both the instrumental, 36 children out of 40, *p* < .001, and the normative condition, 38 children out of 40, *p* < .001, see Fig 7.

### Discussion

In Experiment 2, we explored whether children would choose to copy a consensus versus an expert in two different contexts, instrumental versus normative. Generally, children were equally likely to copy a consensus or a competent individual in both types of context, but most made their choices consistently. Interestingly, these results suggest that children were expressing a personal bias to favour one or the other strategy, rather than randomly choosing a model to copy. We know from prior work that expertise, as well as following the majority, are strong biases, but here individual children judged one source of information or the other to be particularly compelling. Although children were questioned about why they chose a particular method following the study, their responses did not reveal any clear rationale for their choices; see S3 File for classifications and tabulations of these responses.

One might have predicted that children in the normative condition would have favoured copying the majority, since normative actions like naming actions “fepping” or “blicking” are defined by social consensus. As many as 19 of the children chose to copy the model earlier shown to be instrumentally competent, a disposition displayed by both age groups quite similarly.

We found no differences in copying fidelity between normative and instrumental contexts. Similarly to experiment 1, children tended to repeat the modelled action significantly more often than once, perhaps for the same reasons suggested in our discussion of Experiment 1.

## General Discussion

This study is the first to examine whether children choose to copy a competent individual versus a consensus, in both normative and instrumental contexts. Experiment 1 showed that children of all ages are more likely to copy a competent than an incompetent model when learning to complete an object-centered action, in both instrumental and normative contexts. In Experiment 2, when children were faced with two alternative social learning strategies, copying the majority versus a more instrumentally competent expert, responses indicated that both options may be perceived as credible in the age range tested, and importantly, different children tended to choose one of the two options consistently across the two tests employing different target objects. This suggests the tentative conclusion that these children may have developed different preferred learning strategies, although more extended testing will be needed to explore this hypothesis further. However, in neither experiment did the learning context (instrumental or normative) influence copying behavior of one social source rather than the other. In addition, children were more likely to repeat the action they copied more than once, and more often than the two times they saw it performed, in both experiments and across the two learning contexts.

The finding that children copy the more competent of two models when learning about a new skill is consistent with prior research on novel word learning [50–52] and also with a handful of recent studies that have focused instead on action-copying [22,49,53]. However, the causal variables manipulated in these studies differ. For example, Cluver and colleagues [49] found that children may favour copying a model who is a “good helper” (e.g., makes eye contact and gives clear pedagogical cues) over a “bad helper” (e.g., no eye contact and mumbling). In another study, children preferred a model who was competent but unconventional (e.g., opening a jar against their neck) over the more conventional manual but unsuccessful alternative [22]. Together, these studies suggest that very young preschool children are already selective and prefer to copy competent over incompetent individuals.

Results from Experiment 1 also suggest that children in the normative condition generalized the expertise of the instrumentally competent individual and thus transferred this expertise across learning domains (instrumental to normative). This result contributes to an emerging debate over whether children generalize expertise across different domains (a “Halo effect”), or whether they see expertise as domain-specific. Some recent studies suggest that children favour informants who are experts only in a particular sphere of knowledge, such as word labelling, or choosing a doctor versus a mechanic to fix a broken bone or car respectively [54–58]. Other studies, like the present study, have shown that children may assume some generalization of expertise across domains of knowledge [59,60]. For example, 5-year-olds used an individual’s prior accuracy at labelling objects to predict that they would be more prosocial in another task [59]. Further research is thus now merited to examine whether children generalize expertise across a variety of other domains, such as other normative or instrumental tasks and skills.

We also recognise that having an instrumental expert in the history phase may have influenced children to perceive the normative test trials in a different light. Further work is needed to examine how well children understand normative expertise and whether this form of expertise transfers across learning domains. This area of research has the potential to reveal how children understand particular cues to in- and out-group behaviors and whether children choose to trust or copy conventional or non-conventional models across various tasks.

The main focus of the present study was to examine, in Experiment 2, whether children’s selectivity favors a consensus versus an expert when faced with a choice between them, and how this might be influenced by an instrumental versus a normative context. We had predicted that children would choose to copy the expert in a context where there is a specific instrumental goal to achieve, but would be more likely to copy a consensus in a context where the motivation is to learn a local norm [3]. However, we found that children did not choose a strategy differentially based on these contexts. Instead, some children in both contexts chose to follow the consensus and others the competent model, showing a statistically consistent preference in their choice over the two test trials. Thus, the finding of no population-level preferences for one social learning strategy over the other does not imply random choices; to the contrary, as a group these children were indeed demonstrating social learning, but opting for different strategies to do so. We suggest two main questions remain regarding why we did not observe one specific social strategy or the contextual effects predicted. We discuss children’s social strategies first.

Two studies published after our data collection reported evidence that competent or successful models may be preferred over following a majority, but these studies differed in some important aspects from the present study. In one, Wilks and colleagues [35] examined whether 4- and 5-year-old children will choose a majority over an individual in an instrumental context (e.g., opening a puzzle box) but not in conditions where the consensus is unsuccessful and only the individual is successful (see also [6,61] for similar work). In another recent study, Bernard and colleagues [34] examined past reliability and consensus in a normative context (learning object labels). Six- year-olds favored a reliability strategy and trusted either the reliable individual or reliable consensus, but 4- and 5-year-olds endorsed the object labels from the consensus regardless of who had been reliable. Both studies show that in an instrumental context (e.g. opening a puzzle box) or a normative one (e.g. learning a novel word), children preferred the successful individual or successful majority, over unsuccessful individuals and consensuses. The present study differs from this in that we did not pit an incompetent individual against a competent group (nor the reverse: an incompetent consensus with a competent individual). We know from Experiment 1 that children favor the method used by a more competent individual over an incompetent one, and thus we would also predict that a success bias would prevail if we had used a ‘valenced’ majority (conditions using a successful versus unsuccessful majority) rather than a neutral one. However, ooHour current study suggests that when children are presented with a competent individual and a neutral majority (i.e. direct information on success is unavailable, so a child must rely on each model’s ‘track record’), either strategy may be considered a viable source for learning.

We may wonder whether results would be the same in a collectivist culture, where conformist behavior and group harmony are valued more than in relatively individualist cultures like those tested here, where value may more often be placed on personal autonomy and independence. Recent work suggests that children in more collectivist cultures are more likely to employ a majority social learning strategy than children in individualist cultures [5,6,62]. These results differ from studies reviewed above as well those in the adult learning literature [63], which suggest that a success bias tends to prevail over a majority social learning strategy. We are not aware of any study that has examined whether children in a collectivist culture still prefer to follow a majority if they are pitted against a successful individual model. Thus, it would be interesting to see if a success bias prevails in a collectivist culture.

Two additional reasons for why there were no clear group-level biases towards either a majority or a success/expertise social learning strategy could be (i) poor memory (e.g., children could not remember that the previously competent individual could be competent in test trials) or (ii) cognitive load (e.g., that there were too many video demonstrations: 3 videos of the previously successful model doing one action and three videos of the majority models doing an alternative action). We suggest neither reason is likely. We showed that responses in the test trials were not made because of memory difficulties; children were asked at the end of the experiment if they could remember from the history phase which model was the best at getting prizes out of the three puzzles. All but three children remembered who the competent person was in the history phase when asked at the conclusion of the experiment. We also know that their choices were not based on cognitive load; before children were given the puzzle box during the test trials, the experimenter reminded the child what the expert did and what the consensus did. In addition to this reminder and the use of different colored t-shirts, the presentation of the consensus and competent individual were counterbalanced and analyses confirmed that there were no primacy or recency effects. Regardless, children did not perform at chance, but tended to favor one of the two available strategies.

We suggest that a more plausible reason for children preferring either a majority or a success strategy [approximately equally] are that they may reflect individual differences [64]. For example, those children that opted for the method of the majority in both test trials could perhaps reflect higher affiliative motivation [65]. However, we note that the verbal responses we collected do not shed any substantial light on why children of this age chose their particular method (see S2 File and S3 File), and we do not yet know how consistent children would continue to be across time and other contexts. The largest subset of children in both experiments declared they were not sure or aware of why they copied one method over another. Further research could collect additional measures to examine possible correlations between whom children choose to learn from. For example, priming [66], understanding how children understand a group [67], or using measures of affiliation with others, might predict whether a child might be more likely to learn from a majority [28].

Finally, we found that there were no differences between instrumental and normative learning contexts in children’s preferences for the alternative models we tested. A partial explanation for this might be that although we ensured that in the test phase the normative condition did not involve a goal of extracting a reward item from the boxes, but merely acting out a “fepping” or “blicking” act, children had witnessed goal directed, object-centred actions in the history phase. These experiences may have primed an instrumentally-focused perception even in a subsequent normative test, even though itself it had no instrumental goal.

## Conclusion

We suggest the present study makes three major contributions to understanding the ways in which children learn from others. First, it demonstrates that children will reliably copy a model who has been shown to be successful in an action-based rather than verbal task, and that children reliably copy in two different learning contexts (both instrumental and normative) (Experiment 1). This finding contributes to the current debate about whether children generalize expertise to other domains. Second, the study adds to the small but growing literature of how children learn skills and actions selectively (Experiments 1 and 2), complementing the larger existing corpus of language studies [68]. Third, the present study demonstrates that young children as a group are equally likely to copy a competent individual or a majority when learning both instrumental and normative skills (Experiment 2) but that individuals may prefer one strategy consistently. Future work may further explore the individual differences this suggests, and their developmental origins.

## Supporting Information

S1 Dataset Experiment 1. (SAV)

S2 Dataset Experiment 2. (SAV)

S1 File Experimental script, Experiment 1: Establishing competency in normative/instrumental conditions. (PDF)

S2 File Experiment 1, follow-up questions. (PDF)

S3 File Experiment 2, follow-up questions. (PDF)

S4 File Experiment 1, preliminary analyses. (PDF)

S5 File Experimental script, Experiment 2: Do children trust a competent individual or a consensus under normative/instrumental conditions? (PDF)

S1 Table Number of children and their explanations for their chosen method in Experiment 2. (PDF)
