# Supplementary material for: Do Children Copy an Expert or a Majority? Examining Selective Learning in Instrumental and Normative Contexts
Source: PLoS One. 2016 Oct 21;11(10):e0164698. doi: 10.1371/journal.pone.0164698 (PMC5074571; doi:10.1371/journal.pone.0164698)
Supplement: S1 File — (PDF) [file pone.0164698.s003.pdf]

S1 File. Experimental scripts, Experiment 1: Establishing competency in normative/instrumental conditions

Each child sits at a table with a laptop computer in front of them.

Experimenter: "Today, we're going to look at some puzzles I brought in. We are going to watch two people, Miss Yellow and Miss Blue, having a turn with these different puzzles. Shall we say hello to Miss Yellow and Miss Blue?"

[Experimenter directs child to video screen and clicks on either Miss Blue or Miss Yellow- these are counterbalanced].

Experimenter: "Hello, Miss Blue"

Miss Blue: [Miss Blue waves]

Experimenter: "Hello, Miss Yellow"

Miss Yellow: [Miss Yellow waves]

<u>Instrumental History phase:</u>

Experimenter: "Miss Blue and Miss Yellow have three puzzles to show us. In each puzzle there are prizes inside. Let's see if Miss Yellow and Miss Blue know what to do."

[Experimenter clicks either Miss Yellow or Miss Blue] Let's first see Miss Blue/Yellow try to get the prize out.

[video]

Experimenter: Now let's see Miss Yellow/Blue try to get the prize out.

[video]

Experimenter: [next slide-point to each model's picture and say] Did Miss _____ get the prize out or did Miss _____ get the prize out?

[Record response].

If child does not get it right, replay the video. If he/she still does not get it, correct the child].

[repeat the above twice more with 2 additional puzzles]

Instrumental Condition:

Practice box

[Present child with the practice box]

Experimenter: "Now we're going to do something different. Here is a pod. There's only one way to get the prize out. Let's try and figure out which is the way to get the prize. Let's try this way first."

[Tell child to pull out tab on the side and the push back in. Tell her/him to check to see if the prize is in].

Experimenter: "I guess that isn't the way to get the prize. Let's try a different way."

[Tell child to push flap back and forth a few times].

Experimenter: Go ahead and open the box. Is the prize in there?

[Child opens box and retrieves prize. Experimenter puts box away].

Experimenter: "We are now going to see some other pods."

Test Trials

**[Experimenter picks up one of the test trial pods].

Experimenter: "Now we're going to do something different. We can get prizes out of this pod. Like before I'm going to put an egg in here and it should come out of this red box. But, there's only one way to get the prize out. Let's see Miss Yellow and Miss Blue trying to get the prize. First we'll watch Miss Blue/Yellow and then we'll watch Miss Yellow/Blue. Then it will be your turn. Let's watch."

[Experimenter plays video of Miss Yellow/Blue-counterbalanced.]

Experimenter: Let's watch Miss Blue/Yellow trying to get the prize.

[Then, click on other video to watch other model. When video has finished, hand children the box].

Experimenter: "Now it's your turn to get the prize out."

[Record child's response. If child struggles, use agreed upon script for encouragement.'].

Experimenter: "Well done. I know you're dying to know what's inside. Let's wait to open it after we see the second pod. OK?"

[Procedure repeats**]

[After child has done second trial, allow him/her to have the key and open the boxes to retrieve prizes].

After both test trials, Experimenter: "If you remember from a little bit ago when we looked at those puzzles, who was best at getting the prizes?"

[Experiment ends.]

Normative Condition:

Practice box

[Present child with the practice box]

Experimenter: "Now we're going to do something different. Here is a pod. And this pod is for daxing. There's only one way to dax. Let's try and figure out which is the way to dax. Let's try this way first."

[Take rod out and stick it in the side hole. Take egg out].

Experimenter: "Hmmm, I wonder if that was the way to dax. Let's ask these people if that was the way to dax." [Play video of faces shaking their heads and putting thumbs down]. "Hmmm..I guess that wasn't the way to dax. Let's try a different way."

[Push handle back and forth three times and then take egg out].

Experimenter: "I wonder if that was the way to dax. Let's ask these people again." [Play video of faces smiling and putting their thumbs up]. "I guess this is the way to dax." [Wiggle the paddle back and forth].

[Experimenter puts box away].

Experimenter: "We are now going to see some other pods. And you'll get to have a go with them."

Test Trials

**Experimenter: "Now we're going to do something different. Here is a pod. This pod is for fepping/blicking. Let's watch Miss Blue and Miss Yellow trying to fepp/blick. Then it will be your turn. Let's watch."

[Experimenter clicks either Miss Yellow or Miss Blue]

[When video has finished, hand children the box].

Experimenter: “Now it’s your turn to fepp/blick.”

[Record child’s response. If child struggles, use agreed upon script for encouragement.’].

Experimenter: “Well done. We have one more pod.”

[Procedure repeats**]

After both test trials, Experimenter: “If you remember from a little bit ago when we looked at those puzzles, who was best at getting the prizes?”

[Experiment Ends]
