# Supplementary material for: Do Children Copy an Expert or a Majority? Examining Selective Learning in Instrumental and Normative Contexts
Source: PLoS One. 2016 Oct 21;11(10):e0164698. doi: 10.1371/journal.pone.0164698 (PMC5074571; doi:10.1371/journal.pone.0164698)
Supplement: S2 File — (PDF) [file pone.0164698.s004.pdf]

S2 File. Experiment 1, follow-up questions

At the end of the experiment, the experimenter asked each child if they could recall which model was the best at getting prizes out of puzzles from the history phase. Of the 44 children in the study, 43 children correctly recalled which was the competent, expert model. Children's responses for why they chose a certain method were allocated to one of five different types of answers: (1) Functional, or stating how the puzzle box worked; (2) causal, or explaining what happened when a certain method was used; (3) personal preference; (4) mentioning the expertise of the competent model, and (5) "I don't know" responses. Of the 44 children, only 11% said they chose their method based on the competent model (e.g., "Miss Yellow did it this way and she is always right"). Another 9% gave merely functional explanations (e.g., "It gets the egg out."), 20% gave causal explanations (e.g., "Because it [egg] would fall down, and that would push it"), and 16% offered personal preferences for their chosen method (e.g., "I liked this way"). The largest portion of children (44%) said that they did not know why they chose the method they did.
