# Supplementary material for: Do Children Copy an Expert or a Majority? Examining Selective Learning in Instrumental and Normative Contexts
Source: PLoS One. 2016 Oct 21;11(10):e0164698. doi: 10.1371/journal.pone.0164698 (PMC5074571; doi:10.1371/journal.pone.0164698)
Supplement: S3 File — (PDF) [file pone.0164698.s005.pdf]

S3 File. Experiment 2, follow-up questions

Following the experiment, we asked children two questions. We first asked children to recall why they chose each method on each test trial. Responses fell into six different classes (see Table 1). Only a minority of children said their choice was based on the expertise of the model or on the majority preference in the distribution across all six responses, nor was there a significant difference in the contrast between competent versus majority explanations alone (p = .08, fishers exact test). Overall, regardless of the social learning strategy children chose (majority, expert, or ambivalent), 15% of children said they chose their method because of the competent model (e.g., “Because Miss Orange did it this way”), 11% gave causal explanations referring to the workings of the boxes (e.g., “I thought this would slide the egg into the box”), another 16% said they chose the method merely out of stated preference for that method (e.g., “I liked it this way”), 8% gave functional explanations (e.g., this looks like a handle and handles can get prizes”), and just 3% said they chose the method because that was chosen by the majority. The majority of children (49%) said they did not know why they chose the method. Thus, verbal responses cast no significant light on the basis for children’s consistent but different social learning strategies.

In the second follow-up question we asked children to recall which model was the competent model. All but three children correctly recalled who the competent model was from the history phase.
