# Supplementary material for: Do Children Copy an Expert or a Majority? Examining Selective Learning in Instrumental and Normative Contexts
Source: PLoS One. 2016 Oct 21;11(10):e0164698. doi: 10.1371/journal.pone.0164698 (PMC5074571; doi:10.1371/journal.pone.0164698)
Supplement: S4 File — (PDF) [file pone.0164698.s006.pdf]

S4 File. Experiment 1, preliminary analyses

We investigated whether children consistently copied the same model for both test trials or whether children switched strategies to match the method of a different model between the first and second trial. We used nonparametric tests and found that there were no order effects in the instrumental condition, p = .62, and in the normative condition, p = .89. There were also no differences in performance for choosing the competent model by gender in the instrumental, χ^2 (1, N= 22) =.94, p =.64, or normative condition, χ^2 (1, N= 22) =.375, p =.66. Finally, there were no differences in responses across both test boxes across conditions, χ^2 (1, N= 44) =.16, p =.22.
