# Supplementary material for: Do Children Copy an Expert or a Majority? Examining Selective Learning in Instrumental and Normative Contexts
Source: PLoS One. 2016 Oct 21;11(10):e0164698. doi: 10.1371/journal.pone.0164698 (PMC5074571; doi:10.1371/journal.pone.0164698)
Supplement: S5 File — (PDF) [file pone.0164698.s007.pdf]

S5 File. Experimental scripts, Experiment 2: Do children trust a competent individual or a consensus under normative/instrumental conditions?

Each child sits at a table with a laptop computer in front of them.

Experimenter: "Today, we're going to look at some different puzzles that I brought in. We are going to watch five different girls having a turn with these different things. The names of the people are Miss Orange, Miss Blue, Miss Green, Miss Purple and Miss Yellow. Shall we say hello to them?"

[Experimenter directs child to video screen and clicks on either Miss Blue or Miss Orange/Green/Yellow/Purple-these are counterbalanced].

Experimenter: "Hello, Miss Blue"

[Experimenter clicks on Miss Blue and she waves]

[Experimenter says hello to each model and then clicks on her picture and she waves].

Experimenter: "Now that we've met all the girls that are going to join us today, [Miss Blue] and [Miss Orange/ Green/Purple/Yellow] are going to show us some puzzles."

Instrumental History phase:

[Miss Orange/ Green/Purple/Yellow are all counterbalanced]

Experimenter: "[Miss Blue] and [Miss Yellow/Green/Orange/Purple] have three puzzles. In each puzzle there are some prizes inside. Let's see if Miss Orange and Miss Blue know what to do."

[Experimenter clicks either Miss Orange or Miss Blue]

[video]

Experimenter: Now let's see Miss Orange/Blue try to get the prize out.

[video]

Experimenter: [new slide-point to each model's picture and say] Who got the prize?

[Record response].

If child does not get it right, replay the video. If he/she still does not get it, correct the child].

[repeat the above twice more with 2 additional puzzles]

Instrumental Condition:

Practice box

[Present child with the practice box]

Experimenter: "Now we're going to do something different. Here is a pod. There's only one way to get the prize out. Let's try and figure out which is the way to get the prize. Let's try this way first."

[Tell child to pull out tab on the side and the push back in. Tell her/him to check to see if the prize is in].

Experimenter: "I guess that isn't the way to get the prize. Let's try a different way."

Experimenter: Go ahead and open the box. Is the prize in there?

[Child opens box and retrieves prize. Experimenter puts box away].

Experimenter: "We are now going to see some other pods."

Test trials

**[Experimenter picks up one of the test trial pods].

Experimenter: We can get prizes out of this pod. Like before I'm going to put an egg in here and it should come out of this red box. But, there's only one way to get the prize out. Our friends are going to show us what they do when they try and get the prizes out and then it will be your turn. [Show video of four models and experimenter bringing in pod]. Here is Miss Green, Miss Yellow, Miss Orange, and Miss Purple and they all had several goes with this same pod. Is that the same pod as the one I am holding?"

[Competent] Experimenter: Ok. So here is Miss [competent model] and Miss [competent model] had three goes. Let's watch her first go.

[Competent] Experimenter: Here is Miss [competent model]'s second go.

[Competent] Experimenter: Here is Miss [competent model]'s last go.

[Majority] Experimenter: Let's watch the other girls have a go. Now, let's watch Miss [Majority] have a go.

[Majority] Experimenter: And, let's watch Miss [Majority] have a go.

[Majority] Experimenter: Finally, let's watch Miss [Majority] have a go.

[Experimenter plays video of each model getting prize-counterbalanced- half children get Majority models first and the other half of children get Competence Model first.]

[Show slides of each method and remind child what each model did].

Experimenter: "Now it's your turn to try to get the prize out. There's only one way to get the prize out. To remind you, Miss [Competent model] did it this way, on her first, second, and third go, and Miss [model] did it this way, and so did Miss [model] and Miss [model]."

[When video has finished, hand children the box].

Experimenter: "Now it's your turn to try to get the prize out."

[Record child's response. If child struggles, use agreed upon script for encouragement.'].

Experimenter: "Well done. I know you're dying to know what's inside. Let's wait to open it after we see the second pod. OK?"

[Procedure repeats**]

[After child has done second trial, allow him/her to have the key and open the boxes to retrieve prizes].

After both test trials, Experimenter: "If you remember from a little bit ago when we looked at those puzzles, who was best at getting the prizes?"

[Experiment ends.]

<u>Normative Condition:</u>

Practice box

[Present child with the practice box]

Experimenter: "Now we're going to do something different. Here is a pod. And this pod is for daxing. There's only one way to dax. Let's try and figure out which is the way to dax. Let's try this way first."

[Take rod out and stick it in the side hole. Take egg out].

Experimenter: "Hmmm, I wonder if that was the way to dax. Let's ask these people if that was the way to dax." [Play video of faces shaking their heads and putting thumbs down]. "Hmmm..I guess that wasn't the way to dax. Let's try a different way."

[Push handle back and forth three times and then take egg out].

Experimenter: "I wonder if that was the way to dax. Let's ask these people again." [Play video of faces smiling and putting their thumbs up]. "I guess this is the way to dax." [Wiggle the paddle back and forth].

[Experimenter puts box away].

Experimenter: "We are now going to see some other pods. And you'll get to have a go with them."

Test Trials

**[Experimenter picks up one of the test trial pods].

Experimenter: "This pod is for blicking/fepping. There's only one way to blick/fepp. Our friends are going to show us what they do when they try and blick/fepp and then it will be your turn. [Show video of four models and experimenter bringing in pod]. Here is Miss Green, Miss Yellow, Miss Orange, and Miss Purple and they all had several goes with this same pod. Is that the same pod as the one I am holding?"

[Competent] Experimenter: Ok. So here is Miss [competent model] and Miss [competent model] had three goes. Let's watch her first go.

[Competent] Experimenter: Here is Miss [competent model]'s second go.

[Competent] Experimenter: Here is Miss [competent model]'s last go.

[Majority] Experimenter: Let's watch the other girls have a go. Now, let's watch Miss [Majority] have a go.

[Majority] Experimenter: And, let's watch Miss [Majority] have a go.

[Majority] Experimenter: Finally, let's watch Miss [Majority] have a go.

[Experimenter plays video of each model getting prize-counterbalanced- half children get Majority models first and the other half of children get Competence Model first.]

[Show slides of each method and remind child what each model did].

Experimenter: "Now it's your turn to blick/fepp. There's only one way to blick/fepp. To remind you, Miss [Competent model] did it this way, on her first, second, and third go, and Miss [model] did it this way, and so did Miss [model] and Miss [model]."

[When video has finished, hand children the box].

Experimenter: "Now it's your turn to fepp/blick."

[Record child's response. If child struggles, use agreed upon script for encouragement.'].

Experimenter: "Well done. I know you're dying to know what's inside. Let's wait to open it after we see the second pod. OK?"

[Procedure repeats**]

[After child has done second trial, allow him/her to have the key and open the boxes to retrieve prizes].

After both test trials, Experimenter: "If you remember from a little bit ago when we looked at those puzzles, who was best at getting the prizes?"

[Experiment ends.]
