# Supplementary material for: Do Children Copy an Expert or a Majority? Examining Selective Learning in Instrumental and Normative Contexts
Source: PLoS One. 2016 Oct 21;11(10):e0164698. doi: 10.1371/journal.pone.0164698 (PMC5074571; doi:10.1371/journal.pone.0164698)
Supplement: S1 Table — (PDF) [file pone.0164698.s008.pdf]

S1 Table. Number of children and their explanations for their chosen method in experiment 2.

	Explanation					
	Competent Model	Majority	Causal Explanation	Functional Explanation	Preference	I don't know
Copied Majority	1	1	4	3	5	11
Ambivalent	1	1	2	2	4	10
Copied Competent Model	10	0	3	1	4	18
